# Supplementary figures and images for: Expanding standards in viromics: in silico evaluation of dsDNA viral genome identification, classification, and auxiliary metabolic gene curation
Source: PeerJ. 2021 Jun 14;9:e11447. doi: 10.7717/peerj.11447 (PMC8210812; doi:10.7717/peerj.11447)

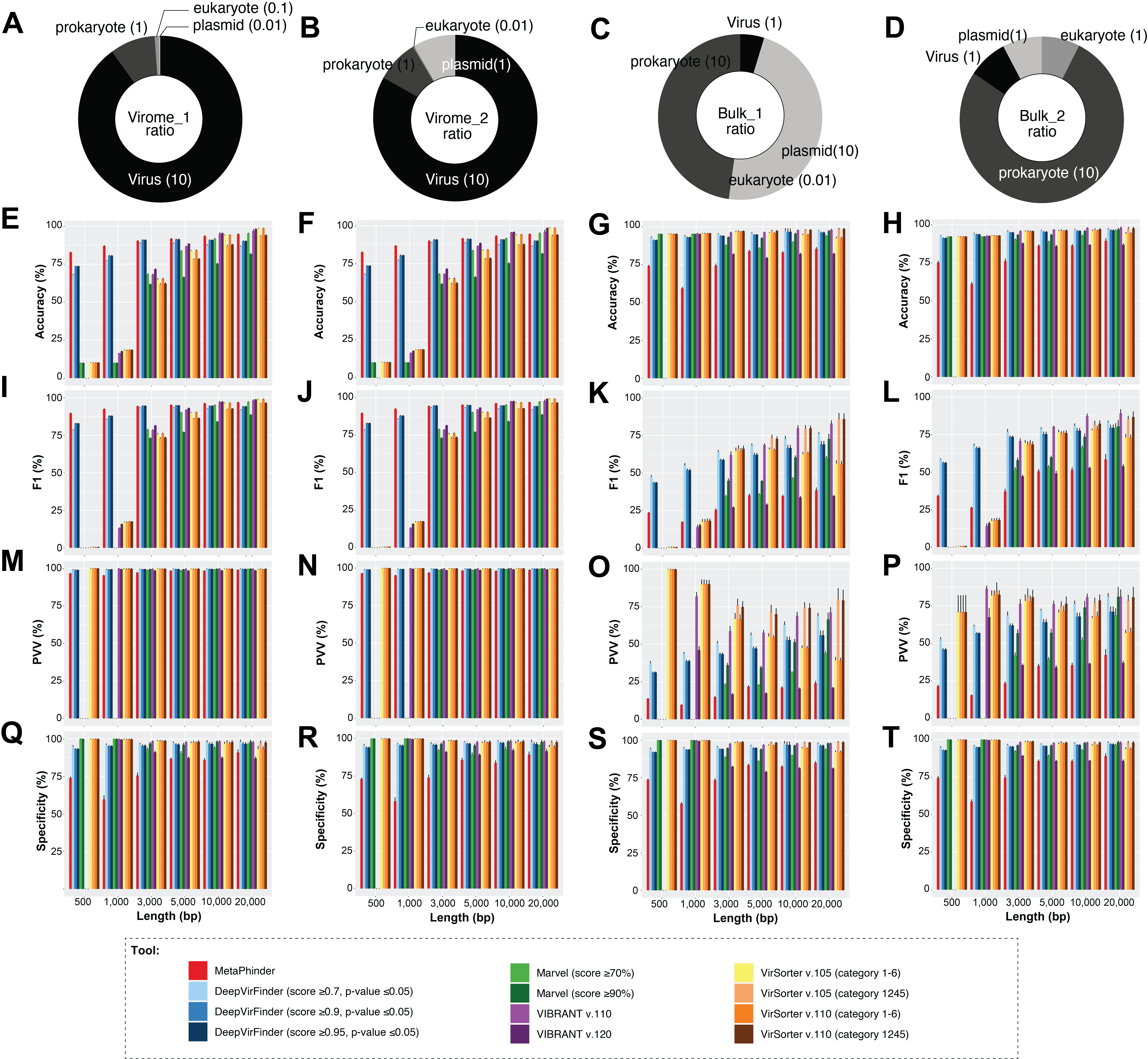

Supplement: Supplemental Information 1 — (A) The viral identification accuracy, (B) F1, (C) PVV, and(D)specificity of DeepVirFinder, MetaPhinder, MARVEL, VIBRANT, and VirSorter on the different fragment sizes of the (i) virome_1, (ii) virome_2, (iii) bulk_1, and (iv) bulk_2 (the composition of the datasets depicted as pie-charts). For DeepVirFinder, three cutoffs were evaluated, i.e., score ≥0.7, ≥0.9, ≥0.95, and p-value ≤0.05. For MARVEL, two cutoffs were used, i.e., scores of ≥70% and ≥90%. Next, we use two different versions of VirSorter, i.e., v1.05 and v1.10, and two cutoffs, i.e., category 1, 2, 3, 4, 5, 6 and category 1, 2, 4, 5. The upper error bars represent the mean of the replicates (see more in Materials and Methods). [file peerj-09-11447-s001.png]

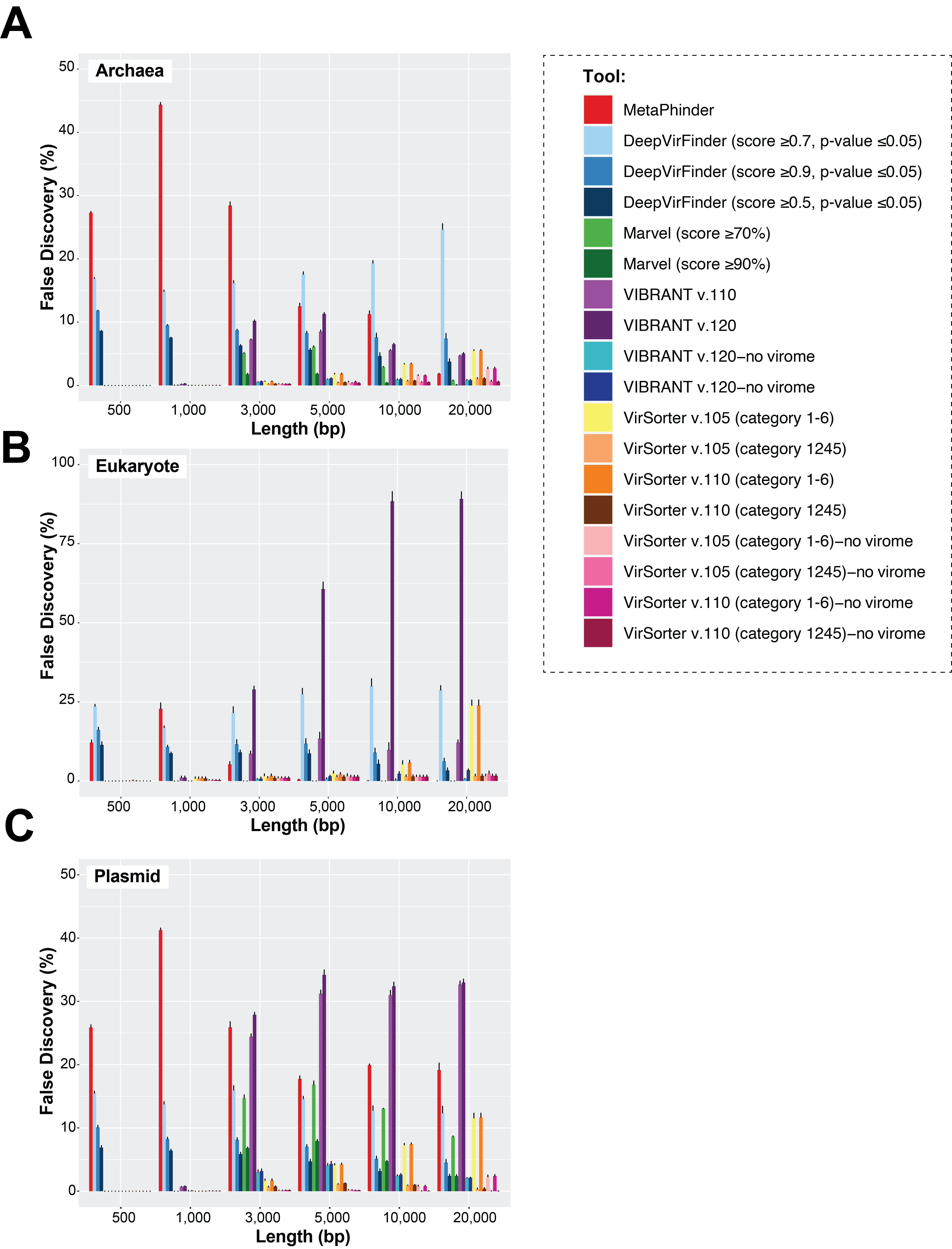

Supplement: Supplemental Information 3 — In addition to the parameters used in (Fig. 2 and Fig. S1), two additional settings of VIBRANT, i.e., without virome flag, were added, and two VirSorter, i.e., without virome flag (standard setting) were also applied. A color gradient represents the viral identification tool. The upper error bars represent the mean of the replicates (see more in Materials and Methods). [file peerj-09-11447-s003.png]

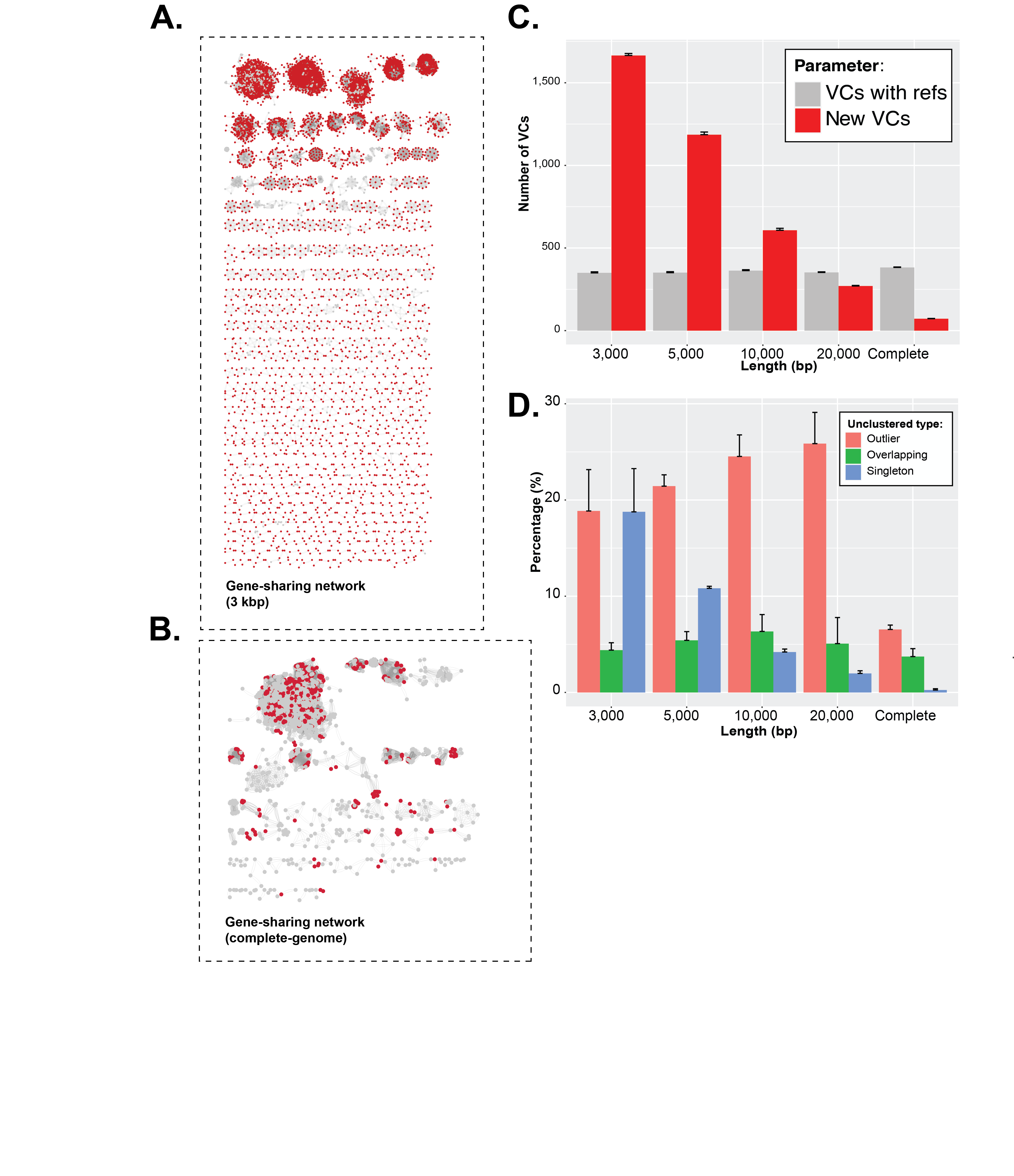

Supplement: Supplemental Information 5 — (A) the gene-sharing network for genome 3 kbp size, and (B) complete-genome. (C) the count of new viral clusters and clusters with reference genomes, and (D) the percentage of the overlapping, outliers, and singletons. [file peerj-09-11447-s005.png]

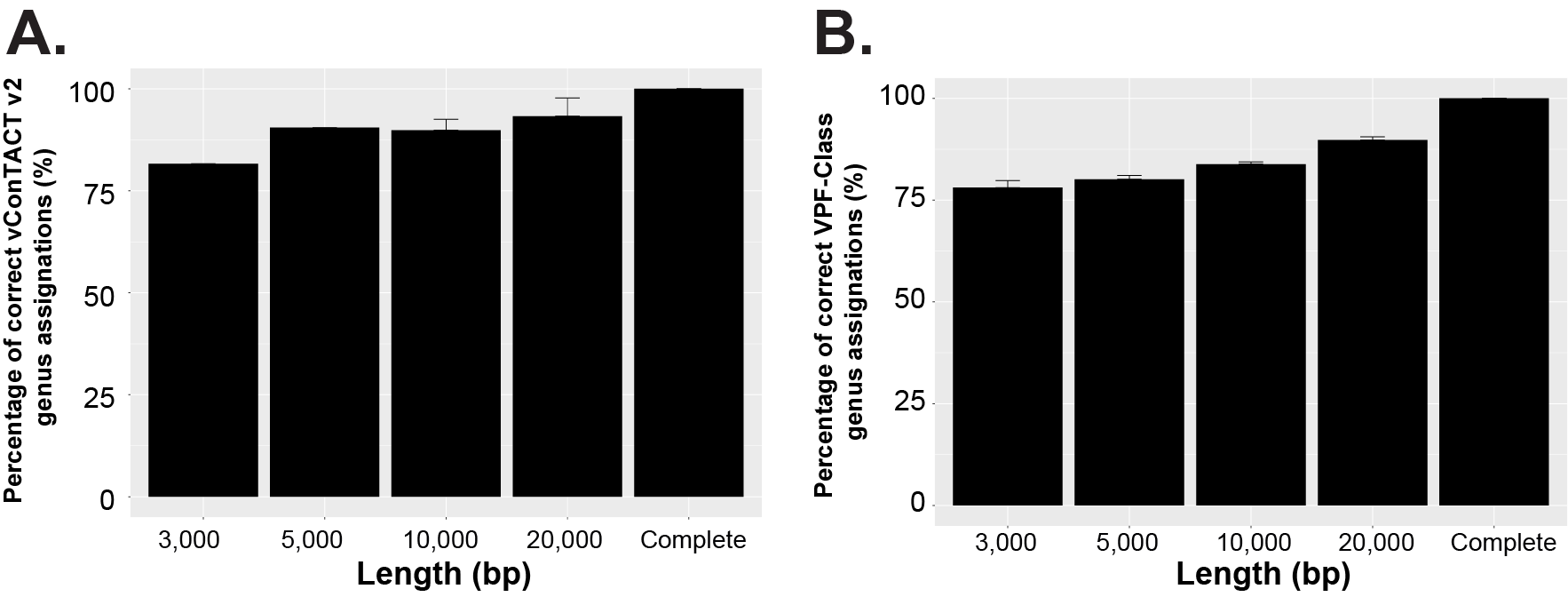

Supplement: Supplemental Information 6 — (A) vConTACT v2 and (B) VFP-class. The analysis was performed by evaluating the correct genus assignment in comparison to the complete genomes. Only the genus affiliated genomes were considered in the evaluation for vConTACT v2. [file peerj-09-11447-s006.png]

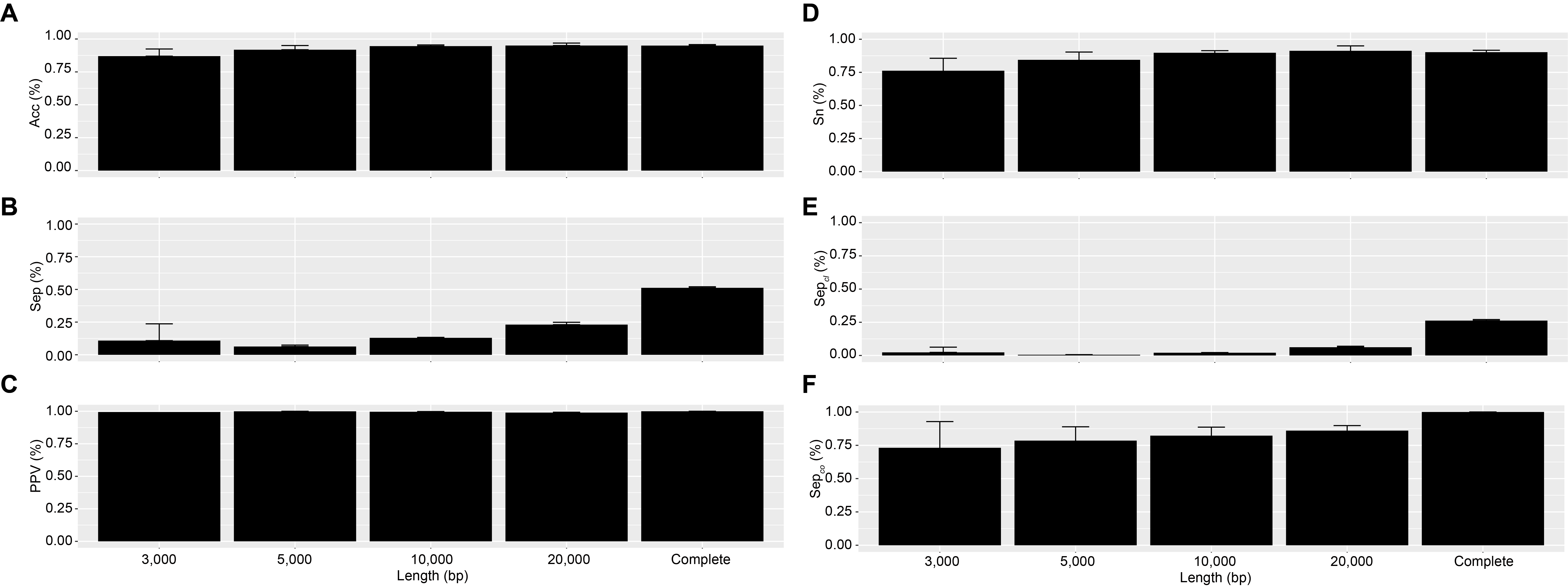

Supplement: Supplemental Information 7 — (A) Accuracy (Acc), (B) Clustering-wise separation (Sep), (C) clustering-wise PPV (PPV), (D) clustering-wise sensitivity (Sn), (E) cluster-wise separation (Sep cl), and (F) complex-wise sensitivity (Sep co). Complete formula can be seen in Jang et al., (2019). [file peerj-09-11447-s007.png]

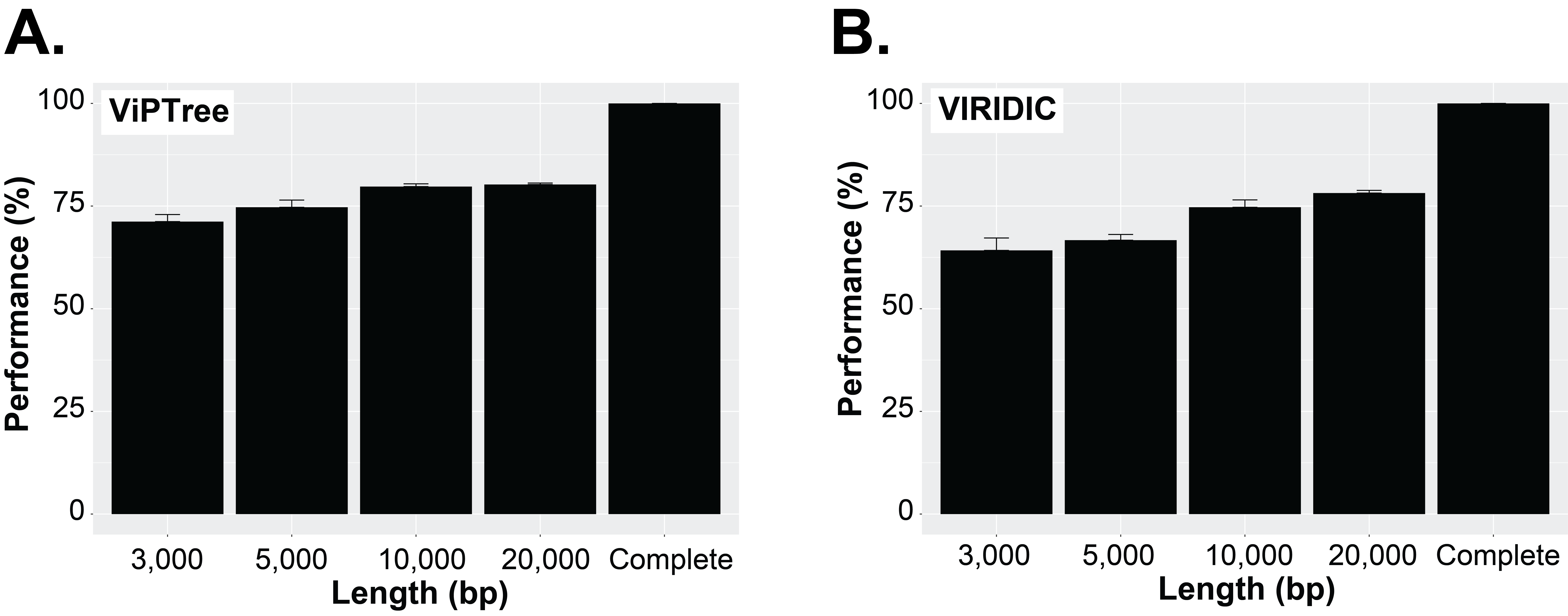

Supplement: Supplemental Information 8 — (A) ViPTree, and (B) VIRIDIC (Nishimura et al., 2017b; Moraru, Varsani & Kropinski, 2020). Because VIRIDIC and ViPTree do not provide a taxonomic affiliation per se but a distance matrix, the differences in performance between different fragment sizes were calculated by comparing the pairwise distances obtained from the fragment sizes with the distances obtained from the complete genomes for the same pair, expressed as percentage relative to the distance obtained from complete genomes. While these percentage reflects differences in the similarity matrix obtained with different fragment sizes for a given tool, these cannot be directly compared between tools or to the performance metrics used to assess the impact of genome fragmentation on the taxonomic classification obtained by vConTACT v2 and VPF-Class. [file peerj-09-11447-s008.png]
